# Supplementary material for: Splice-Junction-Based Mapping of Alternative Isoforms in the Human Proteome
Source: Cell Rep. Author manuscript; Available in PMC 2020 Jan 15. (PMC6961840; doi:10.1016/j.celrep.2019.11.026)
Supplement: 2 [file NIHMS1546469-supplement-2.zip › DF1/PXD006675/CardiacFibroblast/CardiacFibroblast_9_PALLD_EDLLNNGQPEYK.pdf]

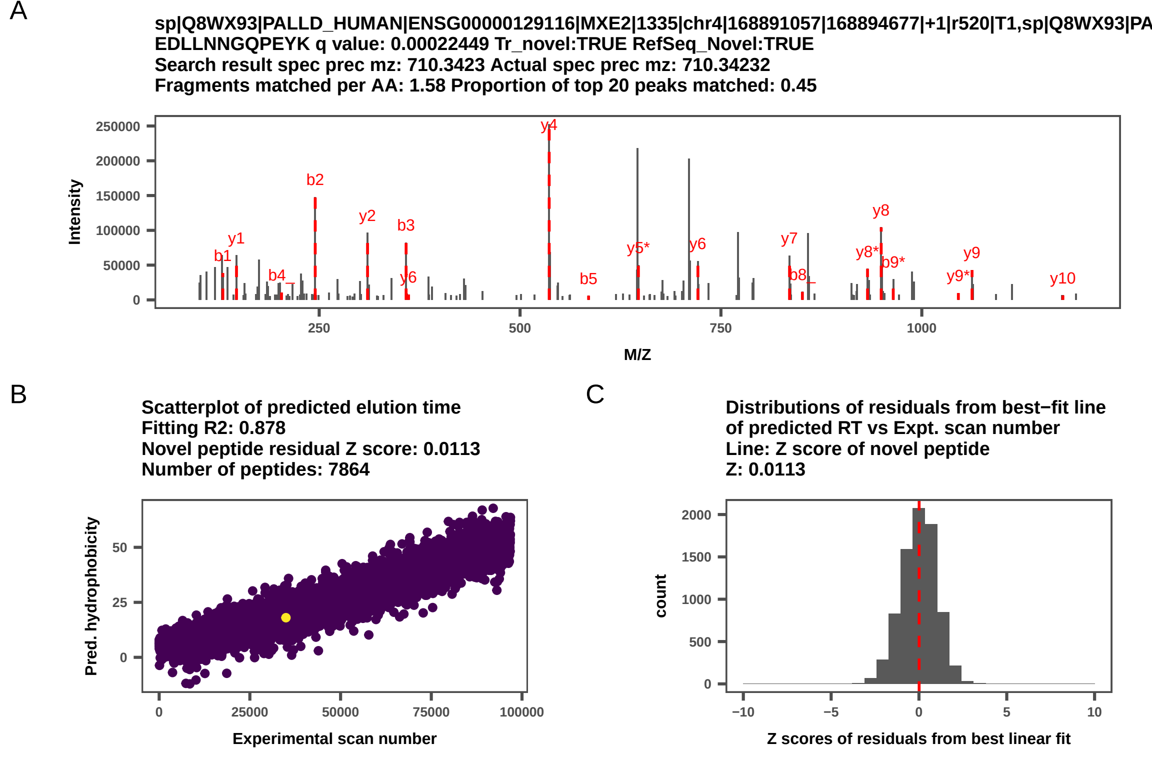
A
sp|Q8WX93|PALLD_HUMAN|ENSG00000129116|MXE2|1335|chr4|168891057|168894677|+1|r520|T1,sp|Q8WX93|PA
EDLLNNGQPEYK q value: 0.00022449 Tr_novel:TRUE RefSeq_Novel:TRUE
Search result spec prec mz: 710.3423 Actual spec prec mz: 710.34232
Fragments matched per AA: 1.58 Proportion of top 20 peaks matched: 0.45
Intensity
M/Z
250000
200000
150000
100000
50000
0
250
500
750
1000
b1
y1
b2
b4_
y2
b3
y6
y4
b5
y5*
y6
y7
b8_
y8*
y8
b9*
y9*
y9
y10
B
Scatterplot of predicted elution time
Fitting R2: 0.878
Novel peptide residual Z score: 0.0113
Number of peptides: 7864
Pred. hydrophobicity
Experimental scan number
50
25
0
0
25000
50000
75000
100000
C
Distributions of residuals from best−fit line
of predicted RT vs Expt. scan number
Line: Z score of novel peptide
Z: 0.0113
count
Z scores of residuals from best linear fit
2000
1500
1000
500
0
−10
−5
0
5
10
